# *HER2 gene assessment* in liquid biopsy of gastric and esophagogastric junction cancer patients qualified for surgery

**DOI:** 10.1186/s12876-020-01531-5

**Published:** 2020-11-16

**Authors:** Anna Grenda, Kamila Wojas-Krawczyk, Tomasz Skoczylas, Paweł Krawczyk, Jadwiga Sierocińska-Sawa, Grzegorz Wallner, Janusz Milanowski

**Affiliations:** 1 Chair and Department of Pneumology, Oncology and Allergology, Medical University of Lublin, Jaczewskiego 8, 20-090 Lublin, Poland; 2 II Chair and Department of General and Gastrointestinal Surgery and Surgical Oncology of the Alimentary Tract, Medical University of Lublin, Staszica 16, 20-080 Lublin, Poland; 3 Laboratory of Pathomorphology, Independent Public Clinical Hospital No. 1 in Lublin, ul. Staszica 11, 20-081 Lublin, Poland

**Keywords:** Gastric cancer, Esophagogastric cancer, *HER2* copy number, Liquid biopsy

## Abstract

**Background:**

Amplification of *HER2* gene (*ERBB2*) and overexpression of HER2 protein on cancer cells are found in 10–26% of gastric cancer (GC) and esophagogastric junction cancer (EGJC). Gene copy number variation (CNV) could be detected in these patients in liquid biopsy and in cancer cells.

**Methods:**

We analysed *HER2* gene CNV used qPCR method in 87 sera collected from GC and EGJC patients before surgical treatment and in 40 sera obtained from healthy donors. *HER2* gene CNV was also assessed in formalin-fixed paraffin-embedded (FFPE) tumor tissue. Furthermore, we assessed the number of *HER2* gene copies and HER2 expression in cancer cells using the fluorescent in situ hybridization method (FISH) and immunohistochemistry (IHC).

**Results:**

We found that the *HER2* gene copy number in liquid biopsy was higher in GC and EGJC patients compared to healthy people (*p* = 0.01). Moreover, EGJC patients had higher number of *HER2* gene copies than healthy donors (*p* = 0.0016). *HER2* CNV examination could distinguish healthy individuals and patients with gastric or esophagogastric junction cancers with sensitivity and specificity of 58% and 98% (AUC = 0.707, 95% CI 0.593–0.821, *p* = 0.004). We found that patients with a high copy number of the *HER2* gene in the tumor tissue assessed by qPCR (but not by FISH) have significantly more often a high number of *HER2* gene copies in liquid biopsy (*p* = 0.04).

**Conclusions:**

We suggested that *HER2* testing in liquid biopsy could be used as an auxiliary method to analysis of HER2 status in tumor tissue in gastric or esophagogastric junction cancers.

## Background

Amplification of *HER2* gene (*ERBB2*) and overexpression of HER2 protein on cancer cells is found in 10–26% of gastric cancer (GC) and esophagogastric junction cancer (EGJC) [1–3]. Approximately 20% of Caucasian patients with metastatic gastric cancer are HER2-positive. Unlike breast cancer, HER2 positivity is not an independent prognostic factor of patients’ outcome in metastatic gastric or EGJC cancers [4]. However, there is evidence that trastuzumab, as neoadjuvant, adjuvant treatment, in combination with oxaliplatin and capecitabine in patients with HER2 overexpressing gastric adenocarcinoma, led to recurrence-free survival of at least 18 months after surgery. Trastuzumab can also be used in combination with oxaliplatin and fluorouracil in the first-line treatment of patients with advanced gastric cancer with high HER2 expression. Patients with *HER2* gene amplification or HER2 protein overexpression may be considered for treatment with molecularly targeted therapy [5, 6]. HER2 protein overexpression is evaluated using immunohistochemistry (IHC). Sometimes confirmation of the IHC result by fluorescent in-situ hybridization (FISH) is required [7, 8]. Despite the methods used for assessment of HER2 status, tests results may be non-diagnostic, due to the nature of the cancer tissue and the difficulties in its proper preparation. There is possibility to use liquid biopsy as a non-invasive method to assess *HER2* amplification in serum or plasma samples [9, 10]. In our study, we attempted to assess the copy number variation (CNV) of the *HER2* gene in circulating free DNA (circulating tumor DNA, ct-DNA) in patients with gastric or esophagogastric junction cancers qualified for surgery.

## Methods

### Patients

We analyzed 87 sera collected from patients before the surgical treatment of gastric cancer (n = 49, 56%) and esophagogastric junction cancer (n = 38, 44%) consecutive diagnosed in II Chair and Department of General and Gastrointestinal Surgery and Surgical Oncology of the Alimentary Tract I Lublin. The control group (compatible in terms of age and sex with the study group) consisted of 40 healthy donors. Moreover we analyzed corresponding available formalin-fixed paraffin-embedded (FFPE) tumor samples (n = 70). The presence of tumor cells in FFPE materials was confirmed by hematoxilin and eosin staining. H&E staining were made on 3 µm sections of paraffin-embedded tissue fixed on Thermo Scientific Superfrost Plus™ glass slides. After staining, slides were assessed by a pathologists, which confirmed the area of neoplastic cells presence.

Forty five (64%) tissue samples were taken before operation and without preoperative treatment and 25 (36%) samples were taken from patients who undergone neoadjuvant treatment (without trastuzumab) before surgery.

The study patients group consisted of 72 men (83%) and 15 women (17%). The median age of cancer patients was 62 ± 8.7 years (range 39–75). Demographic and clinical data are presented in Table 1.

**Table 1:** Demographic and clinical data of cancer patients included in the study

Feature	N (%)	Gastric cancer N = 49 (56%)	Esophagogastric junction cancer N = 38 (44%)	*X*^*2*^ *p*
Age (n = 87)				
< 62	39 (45)	24 (62)	15 (38)	*0.782*
≥ 62	48 (55)	25 (52)	23 (48)	*0.38*
Gender (n = 87)				
Male	72 (83)	41 (57)	31 (43)	*0.066*
Female	15 (17)	8 (53)	7 (47)	*0.8*
Histopathological diagnosis (n = 76)				
Tubular	33 (43)	21 (64)	12 (36)	*12.263*
Mucinous	9 (12)	2 (22)	7 (78)	
Poorly cohesive	20 (26)	13 (65)	7 (35)	
Poorly differentiated	7 (9)	7 (100)	0 (0)	
No data	7 (9)	6 (86)	1 (14)	*0.015*
Laurent type (n = 75)				
Intestinal	21 (28)	17 (81)	4 (19)	*11.802*
Diffuse	15 (20)	13 (87)	2 (13)	
Mixed	7 (9)	4 (57)	3 (43)	
No data	32 (43)	14 (44)	18 (56)	*0.008*
Grading (n = 75)				
G1	3 (4)	1 (33)	2 (67)	*9.391*
G2	33 (44)	16 (48)	17 (52)	
G3	28 (37)	23 (82)	5 (18)	
G4	1 (1)	1 (100)	0 (0)	
No data	10 (13)	7 (70)	3 (30)	*0.052*
cTNM (n = 75)				
IA-IIIA	27 (36)	21 (78)	6 (22)	*3.476*
IIIB-IV	48 (63)	27 (56)	21 (44)	*0.062*
IHC results (n = 19)				
Negative	9 (47)	5 (56)	4 (44)	*0.445*
Low expression of HER2 (+ or ++)	7 (37)	5 (71)	2 (29)	
High expression of HER2 (+++)	3 (16)	2 (67)	1 (33)	*0.8*
Treatment (n = 87)				
PAL	26 (30)	17 (65)	9 (35)	*63.079*
CHTH	3 (3)	3 (100)	0 (0)	
CHRTH	5 (6)	0 (0)	5 (100)	
CHTH + OP	29 (33)	29 (100)	0 (0)	
CHRTH + OP	9 (10)	0 (0)	9 (100)	
OP	15 (17)	0 (0)	15 (100)	*< 0.00000*

*PAL* palliative treatment when primary tumor was inoperable, *CHTH* chemotherapy for gastric cancer, when the tumor was initially assessed as resectable and after neoadjuvant CHTH it was unresectable, *CHRTH* chemoradiotherapy for gastroesophageal junction cancer, when the tumor was initially assessed as resectable and after neoadjuvant CHRTH it was unresectable, *CHTH + OP* for gastric cancer when the combination of preoperative chemotherapy and gastrectomy has been completed, *CHRTH + OP* for gastroesophageal junction cancer when combination of preoperative chemoradiotherapy and esophageal resection has been completed

### DNA extraction

Circulating free DNA (cf-DNA) was isolated from serum samples of patients and healthy individuals with Quick-cfDNA Serum and Plasma DNA Miniprep Kit (Zymo Research, USA) according to manufacturers’ instructions. DNA from FFPE tissues was extracted using QIAamp DNA FFPE Tissue Kit (Qiagen, Germany) according to manufacturers’ instructions. The quality and quantity of DNA was assessed using BioPhotometer UV/Vis Spectrophotometer (Eppendorf, Germany). Samples were stored in − 20 °C until qPCR (quantitative PCR) was performed.

### HER2 CNV assessment by qPCR method

The *HER2* gene copy number was determined by qPCR method. The internal control (housekeeping gene) was the *RNaseP* gene (Applied Biosystems, USA). PCR was performed using Illumina Eco Real-Time PCR equipment (Illumina Inc, San Diego, USA). PCR mixture contained: 5 µl of Genotyping MasterMix (Life Technologies, USA), 2 µl of DNA (5 ng/µl) and 0.5 µl of TaqMan CNV Assay (Hs02428732_cn, Applied Biosystems, USA) or internal control assay, and 3.5 µl of nuclease free water. Following conditions were applied: denaturation and enzyme activation: 95 °C for 10 min, and 40 cycles: 95 °C for 15 s, 62 °C for 2 min. CNV was scored by 2^−ΔΔCt^ method.

### HER2 gene copy number analysis by FISH method

The PathVysion *HER-2* DNA Probe Kit (PathVysion Kit, Abbot Molecular, USA) was used to detect *HER2* gene amplification by fluorescence in situ hybridization technique. Paraffin-Pretreatment and Post-Hybridization Wash Buffer Kit (Abbot Molecular, USA) was also used for the pre-staining procedure. The ProbeChek *HER-2/neu* Normal Signal Ratio Slides (Abbot Molecular, USA) as well as ProbeChek *HER-2/neu* Control Slides (Abbot Molecular, USA) were used for each experiment. The paraffin sections of 3–5 μm thick were cut and mounted on positively charged glass slides. The unstained specimen and control slides were baked overnight at 64 °C. Afterwards, the slides were immersed two times in xylene for 10 min and dehydrated twice in 100% ethanol for 5 min, and then dehydrated in 80% and 70% ethanol for 2 min each at ambient temperature and allowed to dry (app. 2 min). Then, the slides were incubated in 0.2 N HCl for 20 min and washed in purified water for 5 min. After removing excess water from slides, they were incubated for 30 min in Vysis Pretreatment Solution, which had been previously warmed to 80 °C. Afterwards, the slides were incubated in purified water for 3 min and in Vysis Wash Buffer for 10 min. After removing excess wash buffer from slides, they were incubated for 30 min in Vysis Pepsine Solution previously warmed to 37 °C. Then, the slides were washed in Vysis Wash Buffer for 10 min in 4 °C, and in Vysis Wash Buffer for 10 min in room temperature. The slides were placed in a dark room. 10 μl of probe mixture was applied to a slide and immediately covered by a coverslip and sealed with rubber cement. The slides were placed for 5 min on a hotplate at 72 °C and then at 37 °C for overnight hybridization. At the end of the hybridization period, the rubber cement was removed from the slides and they were placed in Wash Buffer at ambient temperature to allow the coverslips to float off the slides. Afterwards, the slides were immersed for 5 min in Wash Buffer previously warmed at 74 °C and air-dried in a dark room. 10 μl of DAPI counterstain was applied to the target area, covered by a coverslip, and the specimens were examined in fluorescence microscope (Nikon Eclipse 55i, Japan).

The PathVysion *HER-2* DNA Probe Kit consists of two labeled DNA probes. The LSI (locus specific identifier) *HER2* probe that spans the entire *HER-2* gene is labeled in Spectrum Orange. The *CEP17* probe is labelled in Spectrum Green and hybridizes to the alpha satellite DNA located at the centromere of chromosome 17 (17p11.1-q11.1). Inclusion of the *CEP17* probe allows for determination of the relative copy number of the *HER2* gene. We determined and recorded the number of LSI *HER-2*/*neu* and *CEP17* counts in 20 nuclei in one region of interest (ROI) and this counting was repeated three times in different ROI. To calculate the final result, we used the following ratio (R): total signals from *HER2* gene locus to total signals from *CEP17* locus. The results were reported as follow: lack of *HER2* gene amplification if the ratio was < 2.2 and presence of *HER2* gene amplification if the ratio was ≥ 2.2 [11].

### Expression of HER2 protein examination by IHC method

Pre-diluted PATHWAY anti-HER-2/neu (clone 4B5) Rabbit Monoclonal Primary Antibody (Ventana, USA) was used for the semi-quantitative detection of HER2 antigen in sections of FFPE tissue on the fully automated VENTANA BenchMark IHC slide staining instrument. IHC staining procedures were performed according to the reagent kit manufacturer's recommendations. UltraView Universal DAB Detection Kit were used as a detection system. Hematoxylin counterstaining was incorporated in the staining protocol. As a negative control rabbit monoclonal negative control immunoglobulin was used (Ventana, USA). A semi-quantitative assessment of HER2 protein expression was performed under light microscopy by two pathologists. Negative results of HER2 expression was in case of no reactivity or membranous reactivity in < 10% of tumour cells. Expression of HER2 1+ was considered for barely perceptible membranous reactivity in > 10% of tumour cells (cells were reactive only in part of their membrane). For equivocal score 2+ was taken weak to moderate complete, basolateral or lateral membranous reactivity in > 10% of tumour cells. Positive HER2 expression 3+ was taken as strong complete, basolateral or lateral membranous reactivity in > 10% of tumour cells.

### Statistical analysis

Statistical analysis was made using Statistica 13.1 software (TIBCO Software, USA). Pearson’s chi-squared test of independence was used whether observations consisting of measures on variables are independent of each other. U-Mann–Whitney test was used to compare medians of analyzed parameters in different groups. Spearman correlation test was used to determine the relationship between particular factors in studied groups. ROC curve (Receiver Operating Characteristic) analysis was made to assess the utility of *HER2* CNV assessment in liquid biopsy as a diagnostic test. The results were considered statistically significant at *p* < 0.05.

## Results

### HER2 gene CNV in liquid biopsy

Seventy-eight samples of liquid biopsy were qualified for analysis. The remaining samples from the 87 pre-qualified tests were rejected due to hemolysis and doubts in the interpretation of the result. We ascertained that *HER2* CNV can be determined in free circulating DNA. The median number of *HER2* gene copies measured by qPCR was 1.85 ± 1.54 (mean 2.1, range 0.18–7.44). 40 (51%) patients had more than 1.85 *HER2* gene copy number and 38 (49%) patients had less than 1.85 *HER2* gene copy number. There were no statistical differences in demographic and clinical factors between groups of patients with high and low number of *HER2* gene copies in liquid biopsy (Table 2).

**Table 2:** Differences in demographic and clinical factors between groups of patients with high and low number of *HER2* gene copies in liquid biopsy

Feature	*HER2* gene copy number in serum		X^2^ *p*
	Below the median N = 38 (49%)	Above the median N = 40 (51%)	
Age (n = 78)			
< 62	17 (50)	17 (50)	*0.04* *0.8*
≥ 62	21 (48)	23 (52)	
Gender (n = 78)			
Male	29 (45)	35 (55)	*1.655* *2.2*
Female	9 (64)	5 (36)	
Histopathological diagnosis (n = 68)			
Tubular	15 (54)	13 (46)	*0.983* *0.9*
Mucinous	5 (64)	3 (36)	
Poorly cohesive	10 (53)	9 (47)	
Poorly differentiated	4 (67)	2 (33)	
No data	3 (43)	4 (57)	
Laurent type (n = 67)			
Intestinal	10 (56)	8 (44)	*3.110* *0.37*
Diffuse	9 (69)	4 (31)	
Mixed	2 (29)	5 (71)	
No data	15 (52)	14 (48)	
Grading (n = 67)			
G1	1 (33)	2 (67)	*2.271* *0.69*
G2	16 (57)	12 (43)	
G3	15 (58)	11 (42)	
G4	0 (0)	1 (100)	
ND	4 (44)	5 (56)	
cTNM (n = 67)			
1A-IIIA	15 (65)	8 (35)	*1.859* *0.17*
IIIB-IV	21 (48)	23 (52)	
IHC (n = 19)			
Negative	4 (44)	3 (56)	*0.686* *0.7*
Low expression of HER2 (+ or ++)	4 (80)	1 (20)	
High expression of HER2 (+++)	2 (67)	1 (33)	
Treatment (n = 78)			
PAL	10 (40)	15 (60)	*5.757* *0.33*
CHTH	0 (0)	3 (100)	
CHRTH	1 (25)	3 (75)	
CHTH + OP	14 (56)	11 (44)	
CHRTH + OP	5 (56)	4 (44)	
OP	5 (42)	7 (58)	

**Fig. 1:**
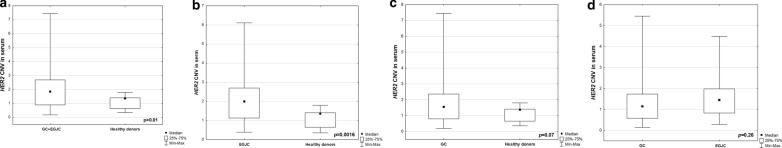
Differences in *HER2* gene copy number detected in sera in patients with gastric or gastroesophageal junction cancer and healthy donors (description in the text)

The analysis showed that the *HER2* gene copy number in liquid biopsy was higher in GC and EGJC patients compared to healthy people (*p* = 0.01, Fig. 1a). Moreover, EGJC patients had higher number of *HER2* gene copies than healthy donors (*p* = 0.0016, Fig. 1b). However, only slightly higher, not statistically significant *HER2* gene copy number were observed in patients with gastric cancer compared to healthy people (median 1.56 vs 1.26, *p* = 0.07, Fig. 1c). There was no statistically significant difference in the *HER2* gene copy number between patients with gastric cancer and with esophagogastric junction cancer (*p* = 0.26, Fig. 1d).

**Fig. 2:**
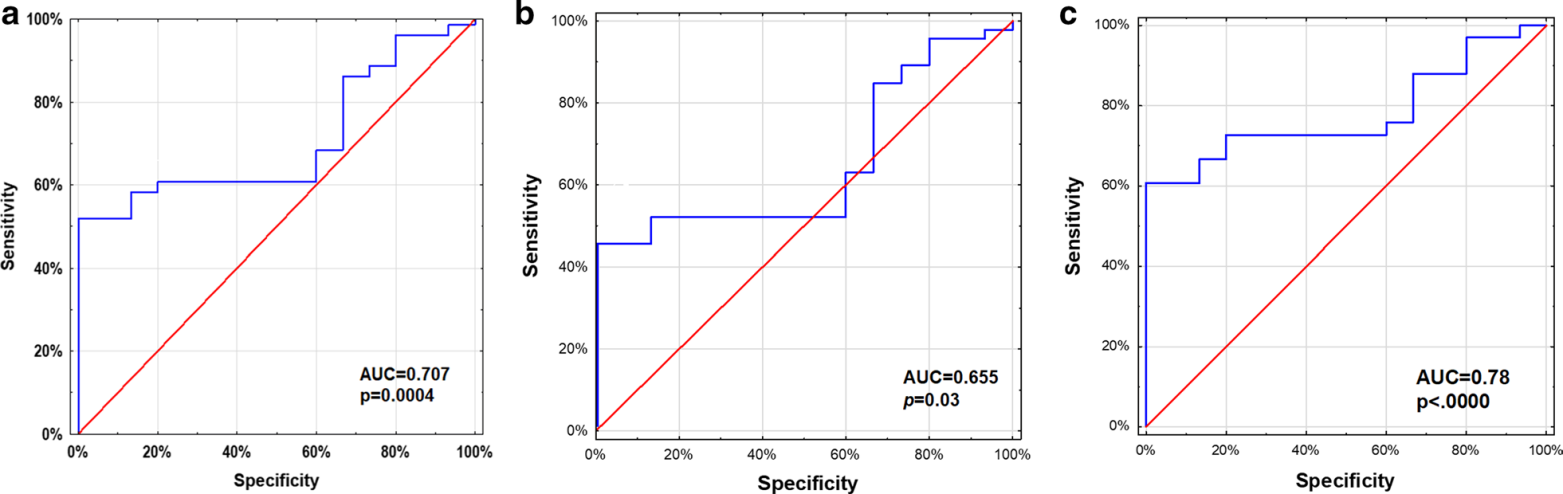
The ROC curve for *HER2* CNV analysis determining the sensitivity and specificity of the test to distinguish cancer patients from healthy people (description in the text)

To assess the usefulness of the *HER2* gene CNV assessment in liquid biopsy as diagnostic test, we performed a ROC analysis with AUC (Area Under Curve) estimation. We found that the sensitivity and specificity of the test to distinguish patients with GC and EGJC from healthy people was 58% and 98%, respectively (AUC = 0.707, 95% CI 0.593–0.821, *p* = 0.004, Fig. 2a). Sensitivity and specificity of the test to distinguish patients with GC and healthy persons was 43% and 100% respectively (AUC = 0.655, 95% CI 0.511–0.799, *p* = 0.03, Fig. 2b). Sensitivity and specificity of the test to distinguish patients with EGJC and healthy persons was 59% and 99% respectively (AUC = 0.79, 95% CI 0.651–0.908, *p* < 0.00001, Fig. 2c).

### HER2 gene status in FISH analysis

**Fig. 3:**
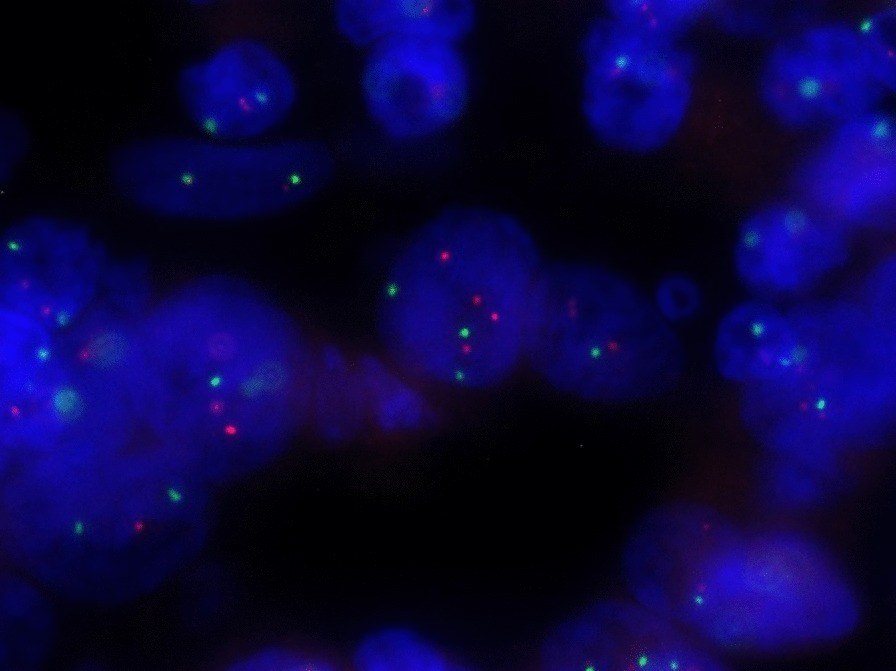
An example of *HER2* gene analysis by FISH method in gastric cancer. Red signals are derived from *HER2* gene locus and green signals are derived from *CEP 17* *locus*

There were 67 (96%) diagnostic materials for FISH analysis among 70 available cases (FFPE tissue samples available). The study group included 37 (55%) GC and 30 (45%) EGJC cases. The mean ratio of total signals from *HER2* gene locus to total signals from *CEP17* locus was 1.96 ± 0.7 (median = 1.76, range 1.5–7.1). The mean number of signals from *HER2* region was 3.26 ± 1.6 (median = 2.78, range 2.03–14.0). Figure 3 shows an example of *HER2* analysis with FISH method in gastric cancer. FISH positive cases, defined as *HER2/CEP17* ratio ≥ 2.2, were detected in 26 (38%) cases, including 14 (38%) positive cases in GC patients’ group and 12 (40%) positive cases in EGJC patients’ group.

The FISH result of the *HER2* gene copy number examination in tumor nuclei did not depend on the demographic and clinical characteristics of the analyzed patients. We did not observe differences in the incidence of different types of GC and EGJC, percentages of patients with high and low expression of HER2 protein, percentages of patients with high and low numbers of *HER2* gene copies in liquid biopsy in groups of patients with or without *HER2* gene amplification in tumor nuclei visualized by FISH method (chi square test, Table 3). We found no significant correlation (Spearman test) between the number of *HER2* gene copies in liquid biopsy and the value of *HER2/CEP17* ratio or numbers of *HER2* gene copies in cancer nuclei observed in FISH method. The number of *HER2* gene copies in liquid biopsy was similar in patients with or without *HER2* gene amplification (U-Mann–Whitney test).

**Table 3:** Tumor type, HER2 protein expression and *HER2* gene copy number in liquid biopsy in patients with or without *HER2* gene amplification in tumor cells visualized by FISH examination

	R < 2.2 N = 41 (62%)	R ≥ 2.2 N = 26 (38%)	X^2^ *p*
Tumor type (n = 67)			
GC patients	23 (62)	14 (38)	*0.033* *0.85*
EGJC patients	18 (60)	12 (40)	
IHC (n = 18)			
Negative	5 (56)	4 (44)	*2.925* *0.23*
Low expression of HER2 (+ or ++)	3 (50)	3 (50)	
High expression of HER2 (+++)	0 (0)	3 (100)	
*HER2* gene copy number in serum (n = 61)			
Below the median	17 (53)	15 (47)	*2.420* *0.12*
Above the median	21 (72)	8 (28)	

The analysis of the relationship between the expression of HER2 protein on tumor cells (IHC method) and *HER2* gene status (FISH method) is unreliable due to the low number of patients who underwent IHC testing. However, we did observe some tendency in this regard. The value of *HER2/CEP17* ratio was statistically significantly higher in patients with high HER2 protein expression than in patients without HER2 protein expression on tumor cells (*p* = 0.04). All patients with high HER2 protein expression also showed *HER2* gene amplification. We found that the number of *HER2* gene copies were slightly higher in patients with high HER2 protein expression compared to patients without HER2 protein expression (*p* = 0.06).

### HER2 gene CNV in tissue measured by qPCR

We assess CNV in FFPE tissue samples in 63 available materials. Median number of *HER2* gene copies measured by qPCR in tissue was 0.95 ± 0.7 (mean = 1.13, range 0.57–4.52). 29 (46%) cancer patients had number of *HER2* gene copies above the median. We found no relationship between the demographic and clinical characteristics of the patients and the number of *HER2* gene copies in tumor tissue assessed by qPCR. We found that statistically significantly more often patients with *HER2* gene copy number above the median in the tumor had also above the median number of *HER2* gene copies in liquid biopsy (chi square test, Table 4). However, the median number of *HER2* gene copies in liquid biopsy in the groups of patients with high (above median) and low (below median) *HER2* gene copy number in the tissue samples (qPCR method) were not statistically significantly different (U-Mann–Whitney test). Moreover, no statistically significant correlation (Spearman test) was found between the *HER2* gene copy number in liquid biopsy and cancer tissue (qPCR method).

**Table 4:** The frequency of coexistence of high and low *HER2* gene copy number depending on the test method and the type of material tested

Feature	*HER2* gene copy number in FFPE tissue measured by qPCR N (%)		X^2^ *p*
	Below the median	Above the median	
*HER2* gene copy number in serum (n = 57)			
Below the median (n = 29, 51%)	19 (66)	10 (34)	*3.939* *0.04*
Above the median (n = 28, 49%)	11 (39)	17 (60)	
*HER2/CEP17* ratio (n = 58)			
R < 2.2 (n = 36, 62%)	20 (56)	16 (44)	*0.07* *0.79*
R ≥ 2.2 (n = 22, 38%)	13 (59)	9 (41)	

**Fig. 4:**
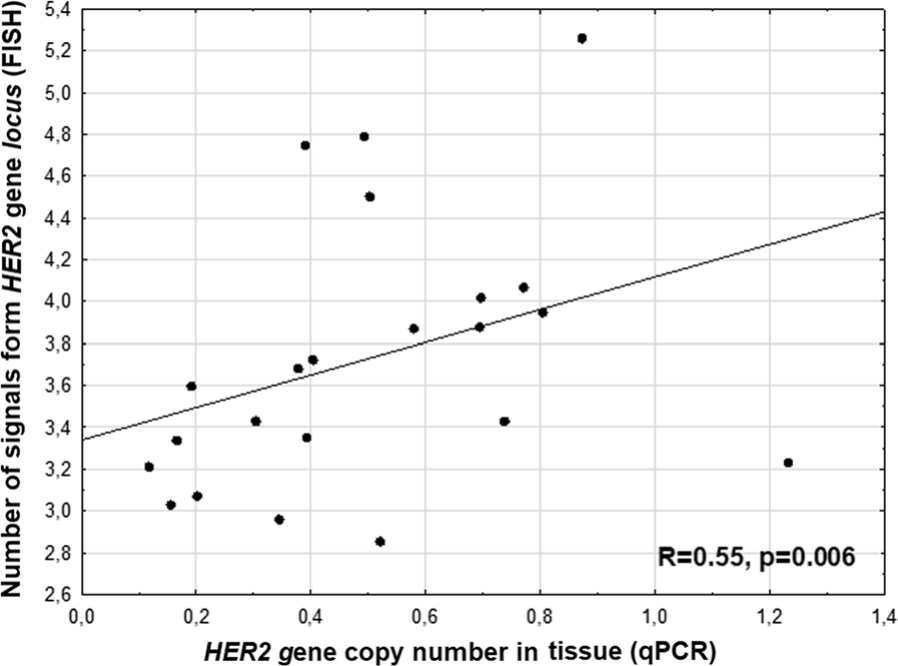
Correlation between HER2 gene copy number in tissue measured by qPCR and number of signals from HER2 gene locus in tumor cell nuclei assessed by FISH method

We detected significant positive correlation between *HER2* gene copy number in tissue measured by qPCR and number of signals from *HER2* gene *locus* in tumor cell nuclei assessed by FISH method (R = 0.55, *p* = 0.006, Fig. 4).

## Discussion

It has been proven that the expression of HER2 on cancer cells in patients with gastrointestial cancers is not such a good predictor of response to anti-HER2 therapy as in patients with breast cancer. However, the need for evaluation of HER2 protein expression or *HER2* gene amplification has become the standard in gastric cancer patients diagnosis. *HER2* overexpression on cells of gastric and esophagogastric junction cancers may affect quarter of the patients. Rajagopal et al. indicated that patients with esophagogastric junction cancer showed HER2 expression in 45.5% of cases. Whereas, only 22.4% of gastric cancer patients showed HER2 expression on cancer cells [12]. The authors presented the results obtained using IHC method.

Unfortunately, only a small fraction of our patients were tested for HER2 protein expression by IHC method before surgery (no access to preoperative treatment with trastuzumab in Poland). However, in most of our patients, we examined the *HER2* gene amplification using the FISH method. This method is used in routine diagnostics to verify questionable results of the IHC test (low expression of HER2 proteins). *HER2* gene amplification is almost always associated with high expression of HER2 protein on tumor cells (we also proved this observation in a small group of patients). We found *HER2* gene amplification in 38% of GC patients and 40% of EGJC patients. Thus, we have not confirmed that *HER2* gene amplification is more common in EGJC patients than in GC patients.

Pyo et al. and Staněk et al. indicated that the compatibility between results of IHC, FISH and qPCR methods performed in tumor tissue is quite high and ranges from 88 to 100% of cases. Nevertheless, it is pointed out that the qPCR results have less compliance with the results of other methods especially in patients with diffuse gastric cancer [13–15]. Also, Zhu et al. found high compliance between IHC and qPCR results in the detection of *HER2* abnormalities in cancerous tissue of gastric cancer. Authors stated that qPCR may be an alternative method of *HER2* testing in tumor tissue [16]. Studies also demonstrated relatively high concordance between elevated serum HER2 level and positive *HER2* status in tumor tissues [17–19].

We detected strong relationship between *HER2* gene copy number in tissue assessed by qPCR and number of *HER2* gene copies in cancer nuclei examined by FISH method. However, we are convinced that quality of FFPE tissue is crucial for obtained reliable results of IHC, FISH and qPCR methods. Therefore, for the first time in the world in GC and EGJC patients qualified for surgery, we have attempted to evaluate the *HER2* gene copy number in cf-DNA in liquid biopsy in patients with GC and EGJC. This method is non-invasive, and enables evaluation of genetic changes in free circulating tumor DNA (ct-DNA), which is usually of good quality. In our study, we did not observe statistically significant correlation between serum and tissue *HER2* gene copy number measured with qPCR but compatibility of results of these tests was 63% (κ = 0.26, data not shown). Therefore, we found that patients with a high copy number of the *HER2* gene in the tumor tissue assessed by qPCR (but not by FISH) had significantly more often a high number of *HER2* gene copies in liquid biopsy. Moreover, GC and EGJC patients had a higher number of *HER2* gene copies in the serum than healthy people. Moreover, we did not notice the difference in *HER2* gene copy number between patients with GC and EGJC. We found that *HER2* CNV examination could distinguish healthy individuals and patients with gastric or esophagogastric junction cancers with sensitivity and specificity of 58% and 98%. The assessment of *HER2* gene copy number in liquid biopsy could supplement IHC and FISH examination. However, we should conclude that it is not possible to replace the diagnosis of HER2 abnormalities in tumor tissue with such diagnosis in liquid biopsy for qualification of patients to anti-HER2 treatment.

Similarly, Sasaki et al. [20] argued that HER2 protein measurement in serum cannot substitute diagnosis of HER2 abnormalities in cancer tissue in advanced gastric cancer patients, despite that they indicated correlation between protein expression in tissue and in serum. They used the chemiluminescent immunoassay test for HER2 detection in serum. However, they have no data regarding the molecular status of *HER2* gene in serum.

Liu et al. [21] studied the plasma *HER2* gene amplification in gastric cancer patients by droplet digital PCR (ddPCR) during 12 months of chemotherapy with fluorouracil and oxaliplatin combined with trastuzumab. They observed that the concordance rate of results of *HER2* gene examination in plasma and FFPE tissue samples by ddPCR was 81.4%, with a sensitivity of 76% and a specificity of 84%. Significant decrease in the plasma *HER2* gene copy number was found after 2 months of treatment [20]. Researchers indicated that serum *HER2* gene assessment may serve as a good marker for monitoring the effectiveness of trastuzumab therapy in patients with gastric cancer. However, authors stated that their study involved only small group of 12 patients.

Kinugasa et al. also used ddPCR to assess *HER2* gene status in ct-DNA in group of 25 gastric patients [22]. They demonstrated that compliance of results of *HER2* gene examination in FFPE tissue samples and in ct-DNA was not high (62.5%). This level of concordance between the results of the *HER2* gene tests in cancer tissue and in liquid biopsy was also found in our study and in study by Kim et al. [9]. In addition Kinuasa et al. found that patients who had high number of *HER2* gene copies in ct-DNA showed significantly shorter survival compared with *HER2*-negative patients [21]. Wang et al. [22] indicated that evaluation of ct-DNA in liquid biopsy by next generation sequencing could be useful in diagnosis of trastuzumab resistance in HER2+ metastatic GC. The median *HER2* gene copy number in ct-DNA in Kinugasa et al. [23] study was 1.15. They assumed the cut-off point above 1.2 gene copy number was considered as *HER2* positivity. Based on this threshold, they found that 7 (29%) out of 24 examined patients were *HER2*-positive. In our study median *HER2* gene copy number was 1.85. Based on this threshold, we found high *HER2* gene copy number in 40 (51%) of 78 GC and EGJC patients. Using tissue material, we detected 26 (38%) of 67 patients with *HER2* gene amplification (FISH method) and 29 (46%) of 63 patients with high number *HER2* gene copies (qPCR method).

There are many more reports on CNV *HER2* evaluation in liquid biopsy in patients with breast cancer compared to GC or EGJC patients [24, 25]. They showed high concordance between *HER2* gene copy number in tissue and in liquid biopsy in breast cancer which is not always observed in the case of gastric cancer and, primarily, in gastroesophageal junction cancer. EGJC is the least studied cancer for *HER2* gene status. Moreover, De Mattos-Arruda indicated that ctDNA from CSF (cerebro-spinal fluid) had the potential to identify brain metastasis-specific actionable genomic alterations that may facilitate the design of personalised treatments in breast cancer patients [26]. There are indication that *HER2* gene copy number can be assessed in serum of peripheral blood as a predictive marker for qualification or monitoring of targeted treatment with trastuzumab in breast cancer patients. Moreover, there are studies on *HER2*-positive free-circulating tumor cells which indicate the possibility of using this diagnostic and monitoring tool in breast cancer patients [27, 28].

In general, a liquid biopsy prior to any treatment implementation can be used as a benchmark for further molecular testing. Due to the lack of reports on the measurement of the *HER2* gene copy number in serum or plasma before surgery in patients with GC or EGJC, further research in this area should be carried out. Before surgery, the correlation between CNV of *HER2* gene in serum or plasma and in tumor tissue is not known and its clinical significance is also unknown. GC or EGJC patients qualified for surgery with a priori analysis of *HER2* gene status could be considered as potential candidates for neoadjuvant treatment with trastuzumab. Clinical trials are conducted with the use of trastuzumab in combination with chemotherapy as adjuvant or neoadjuvant therapy for patients qualified for surgery [29, 30]. These studies use the evaluation of *HER2* gene status in tumor tissue. HER2 expression may change during preoperative treatment. In qualification to postoperative therapy, the ideal solution would be the evaluation of *HER2* gene status in a liquid biopsy. We realize that the introduction of such a method is not easy. However, the availability of material for testing (plasma or serum), without the need to use invasive methods to collect tissue sample, seems to be a great advantage, both in the diagnosis of patients before and after surgery. Further research in this area is definitely necessary.

## Conclusions

Assessment of the copy number of *HER2* gene in GC and EGJC patients’ serum is possible using the qPCR method. Examination of *HER2* gene copy number in liquid biopsy may have some value to supplement the examination of HER2 status in tumor tissue both in patients with GC and EGJC. Survival analysis of patients with known *HER2* CNV status in serum would be very interesting study. Unfortunately, our patients were not subjected to molecularly targeted therapy. The CNV analysis of the *HER2* gene in the serum should be extended in a selected group of patients treated with anti-HER2 antibodies in the context of response to treatment, progression-free survival and overall survival.

## Data Availability

The datasets used and/or analysed during the current study are available from the corresponding author on reasonable request.

## Abbreviations

HER2 (ERBB2): Erb-B2 Receptor Tyrosine Kinase 2
GC: Gastric cancer
EGJC: Esophagogastric junction cancer
FFPE: Formalin-fixed paraffin-embedded tissue
FISH: Fluorescent in situ hybridization method
IHC: Immunohistochemistry
CNV: Copy number variation
cf-DNA: Circulating free DNA
ct-DNA: Circulating tumor DNA
ROC: Receiver operating characteristic
AUC: Area under curve
qPCR: Quantitative polymerase chain reaction
ddPCR: Droplet digital polymerase chain reaction
PAL: Palliative treatment when primary tumor was inoperable
CHTH: Chemotherapy for gastric cancer, when the tumor was initially assessed as resectable and after neoadjuvant CHTH it was unresectable
CHRTH: Chemoradiotherapy for gastroesophageal junction cancer, when the tumor was initially assessed as resectable and after neoadjuvant CHRTH it was unresectable
CHTH + OP: For gastric cancer when the combination of preoperative chemotherapy and gastrectomy has been completed
CHRTH + OP: For gastroesophageal junction cancer when combination of preoperative chemoradiotherapy and esophageal resection has been completed
